# Threshold Microsecond Pulsed Electric Field Exposures for Change in Spinach Quality

**DOI:** 10.1021/acsomega.3c01454

**Published:** 2023-05-24

**Authors:** Zachary Rosenzweig, Abigail Martin, Colin Hackett, Jerrick Garcia, Gary L. Thompson

**Affiliations:** Department of Chemical Engineering, Rowan University, Glassboro, New Jersey 08028, United States

## Abstract

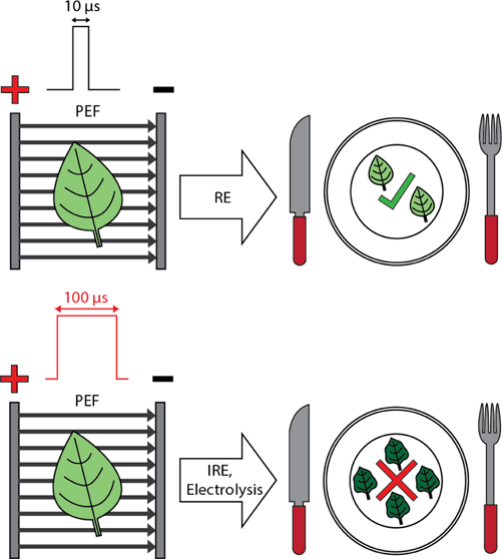

Pulsed electric fields (PEFs) are often used to pretreat foods to enhance subsequent processes, such as drying, where maintaining food product quality is important for consumer satisfaction. This study aims to establish a threshold PEF exposure to determine the doses at which electroporation is viable for use on spinach leaves, wherein integrity is maintained postexposure. Three numbers of consecutive pulses (1, 5, 50) and two pulse durations (10 and 100 μs) have been examined herein at a constant pulse repetition of 10 Hz and 1.4 kV/cm field strength. The data indicate that pore formation in itself is not a cause for loss of spinach leaf food quality, i.e., significant changes in color and water content. Rather, cell death, or the rupture of the cell membrane from a high-intensity treatment, is necessary to significantly alter the exterior integrity of the plant tissue. PEF exposures thus can be used on leafy greens up until the point of inactivation before consumers would see any alterations, making reversible electroporation a viable treatment for consumer-intended products. These results open up future opportunities to use emerging technologies based on PEF exposures and provide useful information in setting parameters to avoid food quality diminishment.

## 1 Introduction

Pulsed electric fields with microsecond durations per pulse (μsPEF) are nonthermal treatments used for food processing. PEF-based electroporation alters the integrity of plant cell walls and membranes, enhancing processes, such as juice extraction and cryopreservation.^1^ PEFs can offer many advantages for juice treatment, including higher and better-quality extraction yields,^2^ and retention and preservation of various vitamins and compounds.^3^ PEF treatment can be combined with other technologies as well. For example, spinach juice treated with both PEFs and ultrasound increased the concentration of several compounds, such as flavonols, carotenoids, and chlorophyll.^4^ Different processes use different mechanisms of electroporation. Pore formation can either be a result of reversible electroporation (RE) or irreversible electroporation (IRE).^5^ RE incurs temporarily enhanced membrane permeability and maintains viability, whereas IRE results in permanent cell death due to the inability to reseal pores and heal the membrane. Several applications utilize RE, such as for enhanced drying^6−8^ or increasing phenolic compounds.^9,10^ These same products are intended to eventually reach consumers, who would expect the same quality, if not better, than unexposed samples.

PEFs are most often used on solid leaves as a pretreatment to enhance another process. For spinach leaves specifically, various processes have been analyzed in combination with PEFs, including vacuum impregnation^11,12^ and hot air drying.^13^ This pretreatment aids in increasing freezing temperatures^11^ and enhancing viability after vacuum impregnation^12^ as well as preserving color and inhibiting shrinkage for convection drying.^13^ This is in addition to enhancing the overall drying process. Although food quality is often reported, many of these papers only determine changes in quality after both the PEF treatment and the subsequent process (e.g., drying) but not from PEFs alone.

When applying PEFs to leafy greens, specifically spinach leaves in this study, it is aimed to treat leaves in such a manner that the external appearance and internal contents remain unchanged. The treatment should preserve color, composition, and overall food product quality. In spinach leaves, water comprises over 90% of the structure by mass^14^ and the green coloration of a leaf indicates freshness.^15^ Water loss and discoloration must be avoided in all stages of food distribution for satisfied consumers.

The improvement of mass transfer from PEFs induces many of the beneficial changes seen in food processing.^16^ So, it may be enticing to increase treatment intensity to induce greater mass transfer. Irreversible electroporation causes a much greater disparity in quality compared to reversible treatments,^17^ with both the pore radius and the number of pores increasing from treatment intensity.^18^ Even in applications where pore formation is reversible, the potential leakage of intracellular content could still affect the makeup of the biological sample, thereby affecting its quality. By applying treatments far below, near, and past the threshold of irreversible electroporation, this work aims to establish a threshold for when quality begins to diminish in solid food processing. Therefore, PEF processing parameters can be selectively chosen to avoid loss of food quality.

## 2 Methods

### 2.1 Raw Material

Semi-savoy spinach leaves (*Spinacia oleracea*) were purchased from a local grocery store (Washington Township, NJ) and used until the listed “Best By” or expiration date. Leaves were stored in a low-density polyethylene storage bag at 4 °C. Only fully intact leaf samples, with no tears and the petiole still attached, were chosen for use. Respective tests were conducted on the same day from the same bag of spinach to minimize differences in initial quality.

### 2.2 Electrical Exposures

**Figure 1:**
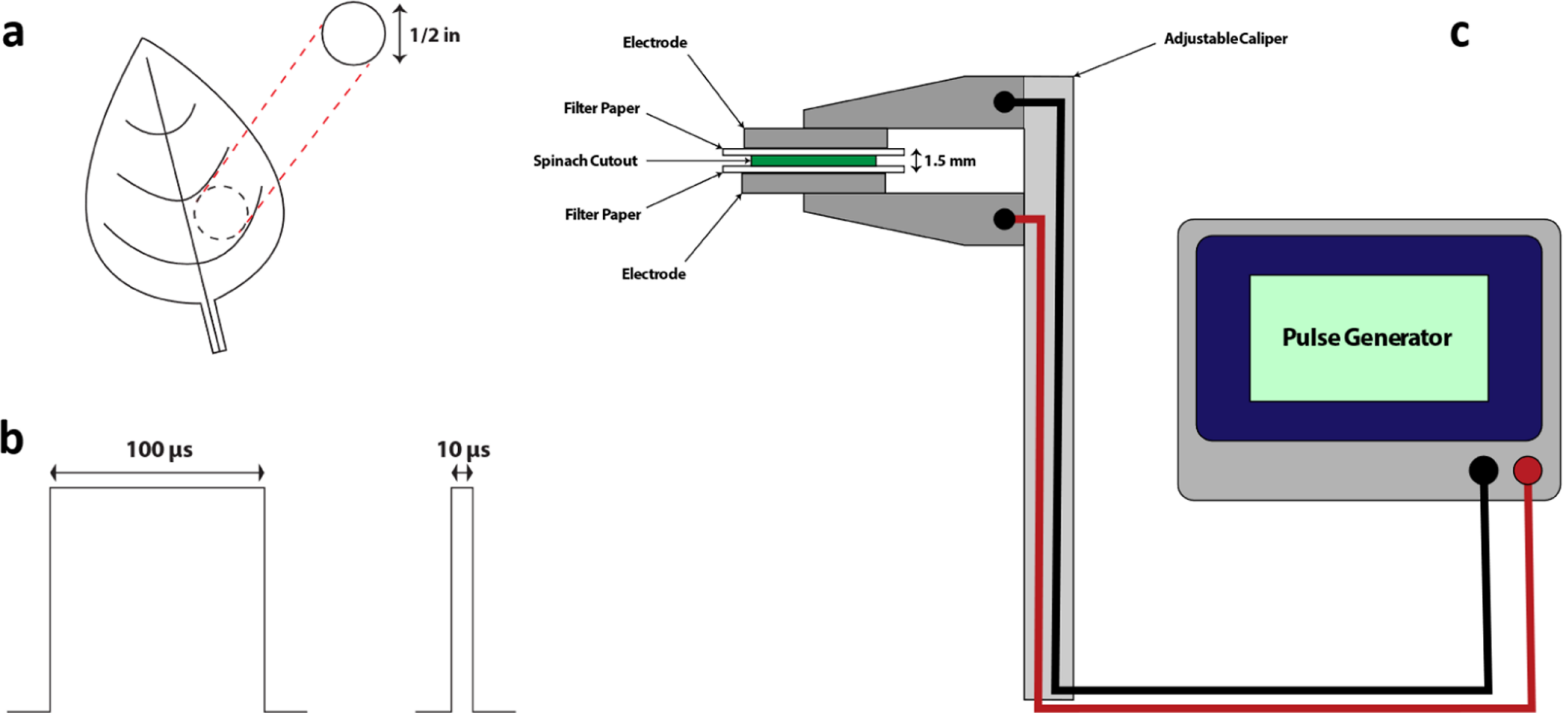
Cutout of spinach leaf (a), pulse shape (b), and setup for pulse delivery (c). Electrodes are about 1.5 cm × 1.5 cm (2.25 cm^2^) compared to the 1.27 cm diameter leaf cutouts (1.27 cm^2^) so samples are completely covered by electrodes. The electrodes are attached to a caliper to set the gap size and are connected to a commercial pulse generator. Images were made using Adobe Illustrator (San Jose, CA).

Leaves were washed with deionized (DI) water. A hole punch was used to cut 1/2″ diameter circles from the spinach (Figure 1), avoiding the midrib and large veins to maintain consistent thickness and weight. The cuts were soaked in a 20 mM Gomori buffer for at least half an hour before use. The buffer had a pH of 7.2, measured using a Mettler Toledo S220 SevenCompact pH meter (Toledo, OH), and a conductivity (σ) of 3.25 mS/cm, measured using a Symphony SP70C handheld conductivity meter (VWR, Radnor, PA). Whatman grade 1 qualitative filter papers (Florham Park, NJ) were cut into squares at least the size of the electrodes (1.5 cm × 1.5 cm) and soaked under the same conditions as the leaves.

A leaf cutout was placed in the middle of two pieces of filter paper and sandwiched between stainless steel (S.S.) Model 384L caliper electrodes (BTX, Holliston, MA), as depicted in Figure 1. Monopolar square wave pulses were delivered using a Gemini X2 electroporation system (BTX, Holliston, MA). Three pulse numbers (1, 5, and 50) and two pulse durations (10 μs and 100 μs) were assessed at a constant 1.4 kV/cm and 10 Hz pulse repetition rate. PEF-treated samples were compared to sham, untreated control samples. Controls were handled and measured the same as treated samples, including resistance measurement with the electroporation system, except no PEFs were applied. The relative timing of measurements for controls was the same as for treated samples.

Total specific energy input was calculated using eq 1
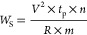
1where *W*_S_ is the total specific energy input (kJ/kg), *V* is the applied voltage (kV), *t*_p_ is the pulse duration (s), *n* is the number of pulses, *R* is the measured resistance (Ω), and *m* is the mass of the sample (kg). A resistance of 75 Ω was used in all calculations based on average readings from the Gemini X2 Pulser, and a mass of 30 mg was used based on the average weight of 10 samples. Calculated values are listed in Table 1.

**Table 1:** Total Specific Energy Input Calculated for Each Parameter

	1P × 10 μs	1P × 100 μs	5P × 10 μs	5P × 100 μs	50P × 10 μs	50P × 100 μs
specific energy (kJ/kg)	0.18	1.78	0.89	8.89	8.89	88.89

### 2.3 Electrochemical Impedance Spectroscopy

Electrochemical impedance spectroscopy (EIS) measurements were acquired using an Interface 1010E potentiostat (Gamry Instruments, Warminster, PA). Impedance was measured over a frequency (ω) range of 100 Hz to 1 MHz directly before and after pulsing, using the same electrode setup used for the exposures. Total impedance, or modulus (*Z*_mod_), ratios were plotted in accordance with eq 2
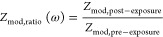
2where *Z*_mod,postexposure_ and *Z*_mod,pre-exposure_ are both functions of ω. The limitations of using a two-electrode setup for EIS measurements are addressed further in Section 4. Five readings were collected for each parameter, aside from the control, which was performed in triplicate.

#### 2.3.1 Equivalent Circuit Model

**Figure 2:**
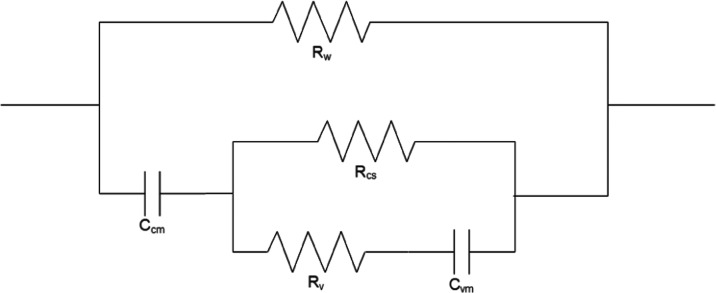
Double-shell model. The resistances of the cell wall/membrane (*R*_w_), cytoplasm/symplasm (*R*_cs_), and the vacuole (*R*_v_), and capacitances of the cell membrane (*C*_cm_) and vacuolar membrane (*C*_vm_) are all represented. This schematic was made using Wondershare (Hong Kong SAR) EdrawMax (v. 11.1.1).

The double-shell model, as described in Jócsák et al.^19^ and diagrammed in Figure 2, was used to determine the values of the resistive and capacitive components of the plant cells being tested. This model accounts for the electrical resistances of the cell wall/membrane (*R*_w_), cytoplasm/symplasm (*R*_cs_), and vacuole (*R*_v_), and the capacitances of the plasma membrane (*C*_cm_) and vacuolar membrane (*C*_vm_).^19,20^ The double-shell model was chosen in this study because of its high conformity for other plant tissues.^20^ Impedance data were analyzed using EChem Analyst 2 (v. 7.10.0.12461, Gamry Instruments).

### 2.4 Cell Staining

Cells were stained using fluorescein diacetate (FDA), using the method described by Dymek et al.,^21^ with some modifications. FDA requires esterases of metabolically active cells to hydrolyze to fluorescein, which can be detected by fluorescence microscopy.^22^ A 350 μM stock solution of FDA and acetone was stored at 4 °C. Immediately before the experiment, the stock solution was diluted to 1.2 nM using DI water. Three cutouts were exposed for each parameter, washed with DI water, and dried using paper towels. A waiting period of at least 15 min was observed before staining to ensure that all reversible pores had closed. Samples were then submerged in the diluted FDA solution for 30 min in the dark and then were washed with DI water and dried thereafter.

Three images from each replicate were captured using an Olympus CKX53 fluorescence microscope (Center Valley, PA) and a PL-D752MU-T camera (Pixelink, Ottawa, ON, Canada) at a constant 15 ms exposure time. Fluorescence intensity of images was analyzed using Fiji (ImageJ2, v. 1.53t) by measuring the mean grayscale value of overall images. The bottom, abaxial surfaces of leaves were imaged for each sample using a 10× objective with 0.25 numerical aperture (NA) (Olympus CACHN10XIPC).

### 2.5 Dry Mass Analysis

To monitor water loss, spinach sections were weighed before and after exposure using a Practum 513–1S precision top-loading balance (Wilmington, DE). Samples were washed with DI water and dried completely of liquid using a Kimwipe (Kimberly-Clark Professional, Chester, PA) before weighing.

### 2.6 Color Analysis

Hunter L*a*b* values were measured using a Chroma Meter CR-100 colorimeter (Konica Minolta, Ramsey, NJ). The colorimeter was calibrated immediately before testing in accordance with the operating manual. L* represents luminance or “lightness” on a black (more negative) to white (more positive) spectrum, a* scales greenness (negative) to redness (positive values), and b* expresses blueness (negative) to yellowness (positive).^23^ For the spinach leaves, only the L* and a* measurements were analyzed. The cutouts were placed on a flat, clean measuring board for readings. Cutouts were larger than the sensor and completely covered it. Measurements were taken before and after exposure, being dried of liquid using the same protocol in Section 2.5. The top adaxial surface of the cutouts, which appeared darker to the naked eye, was analyzed.

### 2.7 pH Analysis

The Fluval pH wide range test kit (Mansfield, MA) was used to measure pH, which includes a colorimetric indicator solution consisting of bromothymol blue, thymol blue, and methyl red. After PEF exposure, 1.5 μL of the indicator solution was added to the filter papers from both the anode and cathode. Images were captured using a 12 MP camera with a *f*/1.6 aperture.

Three random hues were extracted from each image in Adobe Photoshop (v 24.0.1, San Jose, CA) using the eyedropper tool, which were used to convert pH into a standard curve. The standard curve was formulated by measuring hue from color changes on the filter paper at nine different known pH values. A linear relationship exists between hue and pH for this kit and is denoted in eq 3
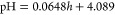
3where *h* represents hue. This curve had an *R*^2^ of 0.978 and is represented in Figure S1. Representative images of the filter paper are shown in Figure S2.

### 2.8 COMSOL Modeling

COMSOL Multiphysics ver. 5.6 (Burlington, MA) is a finite element software that was employed to numerically determine temperature change between the electrodes using the electric current and heat transfer in solid Multiphysics coupling.

Temperature change would be caused by Joule, or ohmic, heating, where the flow of electric current creates thermal energy, and is represented by the following equation in COMSOL
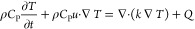
4where ρ is the density (kg/m^3^), *C*_p_ is the specific heat capacity (J/kg/K), *T* is the temperature (K), *u* is the displacement vector, *k* is the thermal conductivity (W/m/K), and *Q* is the heat load (W/m^3^). *Q* can be calculated by eqs 5–7
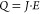
5
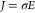
6
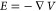
7where *J* is the current density (A/m^2^), *E* is the electric field (V/m), and *V* is the applied voltage.

The model was simplified to assume that only Gomori buffer was present between the electrodes. The buffer was assumed to have the same properties of water, aside from electrical conductivity, which was inputted as 0.33 S/m. Stainless steel electrodes were given the following properties: *k*_ss_ of 15 W/(m·K), ρ_ss_ of 7500 kg/m^3^, *C*_p,ss_ of 465.2 J/(kg·K), σ_ss_ of 1.74 MS/m, and a relative permittivity of 1.0. Only the highest intensity parameter was computed in order to determine the maximum temperature change. An extremely fine free triangular mesh was used at the electrode interfaces and normal everywhere else.

### 2.9 Statistical Analysis

Many of the data sets analyzed were based on changes in spinach leaf properties, postexposure relative to pre-exposure. All differences calculated in this study were based on eq 8
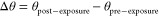
8where θ can represent any variable. Microsoft (Redmond, WA) Excel (ver. 16.63.1) was used to calculate means, confidence intervals, and outliers. GraphPad (San Diego, CA) Prism 9.4.0 was used to run one-way analysis of variance (ANOVA) tests and create graphs. Analyses were performed with five replicates, unless otherwise stated.

## 3 Results

### 3.1 Electrochemical Properties

**Figure 3:**
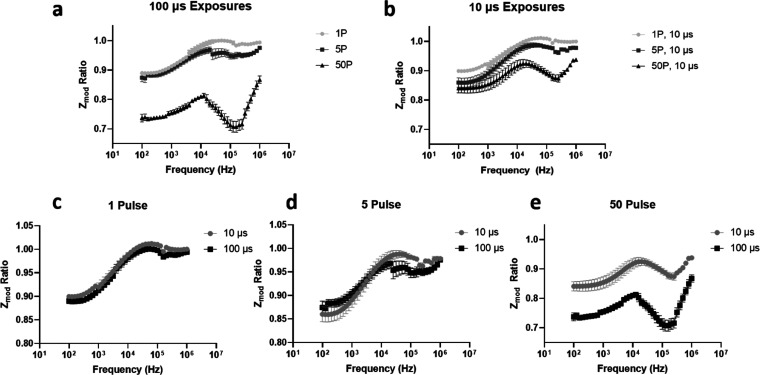
*Z*_mod_ ratios from 100 Hz to 1 MHz for (a) varying pulse numbers for 100 μs exposures, (b) varying pulse numbers for 10 μs exposures, (c) varying pulse durations for the 1 pulse condition, (d) varying pulse durations for the 5-pulse condition, and (e) varying pulse durations for the 50-pulse condition. Error bars indicate the standard error of the mean.

*Z*_mod_ ratios are plotted in Figure 3 and indicate the extent of pore formation among the spinach leaf cutouts. In general, higher intensity exposures induce greater changes in *Z*_mod_ after PEF exposure, with lower ratio values occurring with increasing pulse number and duration. Larger differences occur at the lower end of the frequency spectrum as well.

From the double-shell model, the change in resistances from before to after exposure increases with pulse number and duration. Much greater changes are seen in the resistance of the cell membrane compared to both the vacuolar and cytoplasmic compartments. The same trends are observed for the modeled capacitances. Numerical values for resistances and capacitances can be found in Table 2 and are graphically shown in Figure S3. The average “goodness of fit” of the model to the recorded data is 0.0173, which indicates about a 10% error. Cole–Cole and phase angle plots before and after PEF exposures are represented in Figures S4 and S5, respectively.

**Table 2:** Differences in Resistances and Capacitances for the Double-Shell Model after Exposure at Each Condition with 95% Confidence Intervals

	Δ*C*_cm_ (F)	Δ*C*_vm_ (F)	Δ*R*_w_ (Ω)	Δ*R*_v_ (Ω)	Δ*R*_cs_ (Ω)
control	1.4 × 10^–8^ ± 3.8 × 10^–8^	–8.8 × 10^–10^ ± 1.5 × 10^–9^	–2.9 ± 1.5	0.2 ± 1.4	–3.4 ± 0.2
1P × 10 μs	8.9 × 10^–8^ ± 1.4 × 10^–8^	–3.4 × 10^–9^ ± 3.8 × 10^–9^	–25.8 ± 5.2	–0.6 ± 2.6	2.4 ± 6.4
1P × 100 μs	1.4 × 10^–7^ ± 4.8 × 10^–8^	–2.0 × 10^–9^ ± 1.8 × 10^–9^	–24.9 ± 1.8	–1.8 ± 1.4	2.3 ± 1.1
5P × 10 μs	1.8 × 10^–7^ ± 1.0 × 10^–7^	–1.6 × 10^–9^ ± 3.2 × 10^–9^	–33.2 ± 12.3	–3.5 ± 2.3	1.7 ± 7.5
5P × 100 μs	2.3 × 10^–7^ ± 1.1 × 10^–7^	1.0 × 10^–9^ ± 2.8 × 10^–9^	–23.4 ± 4.8	–3.7 ± 1.3	1.1 ± 3.9
50P × 10 μs	2.4 × 10^–7^ ± 7.8 × 10^–8^	5.1 × 10^–9^ ± 3.0 × 10^–9^	–35.0 ± 6.5	–8.7 ± 4.0	–2.8 ± 10.2
50P × 100 μs	3.1 × 10^–7^ ± 2.8 × 10^–7^	2.0 × 10^–8^ ± 8.7 × 10^–9^	–76.3 ± 39.5	–16.8 ± 13.3	–17.7 ± 13.3

### 3.2 Mass Analysis

**Figure 4:**
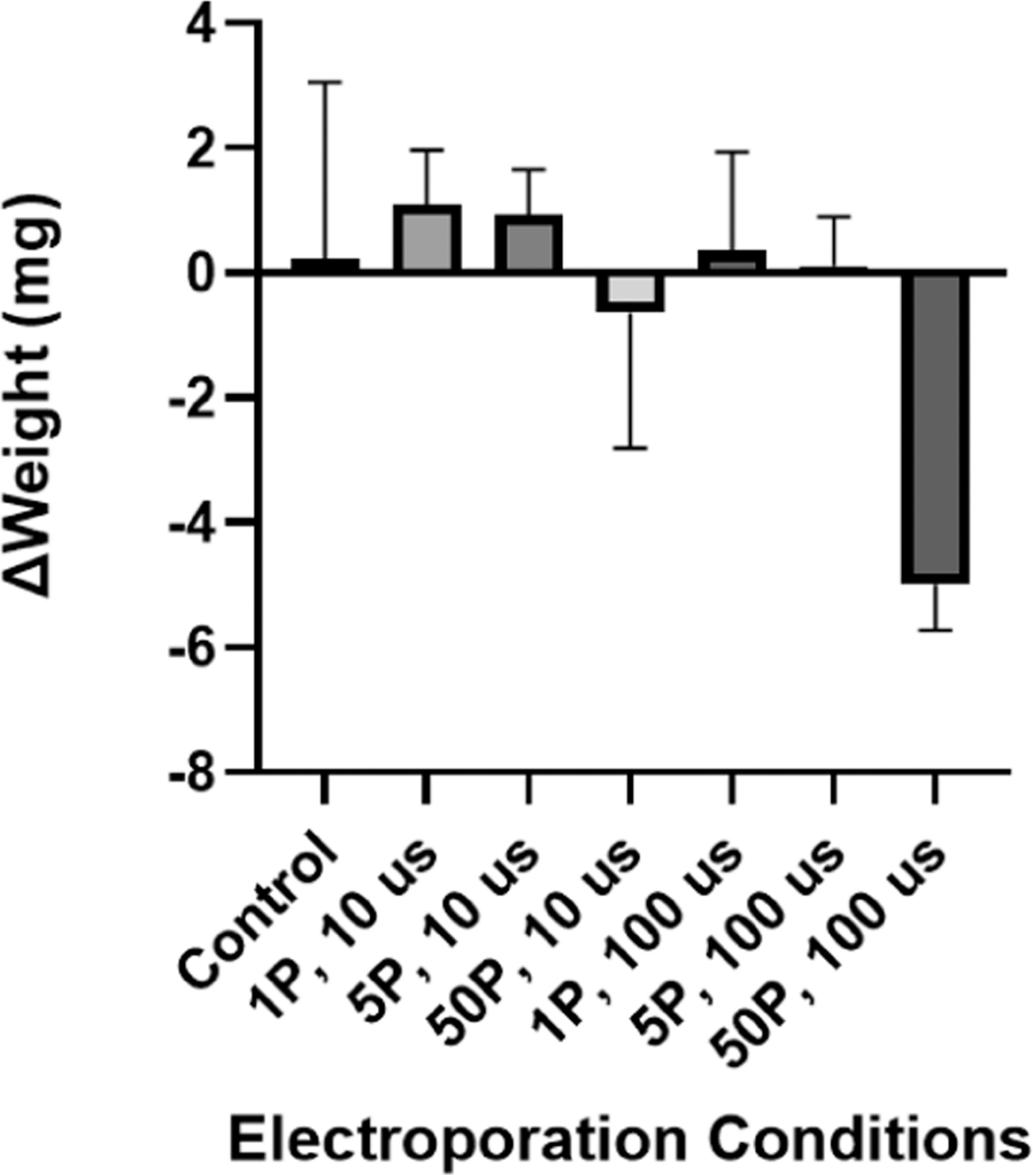
Differences in weight after exposure for each parameter. Statistical significance from the one-way ANOVA results is represented as (ns) ≥0.05, *P ≤ 0.05, **P ≤ 0.01, ***P ≤ 0.001, ****P ≤ 0.0001. Error bars indicate 95% confidence intervals.

A significant change in the mass of treated samples compared to the untreated samples is only seen at the 50P × 100 μs condition (Figure 4). Cutouts weighed 31.9 ± 3.4 mg at a 95% confidence interval before exposure. The controls display an average change in weight of only 0.2 ± 2.8 mg at a 95% confidence interval, whereas the 50P × 100 μs treatment samples display an average change of −5.0 ± 0.7 mg at a 95% confidence interval.

### 3.3 Color Changes

**Figure 5:**
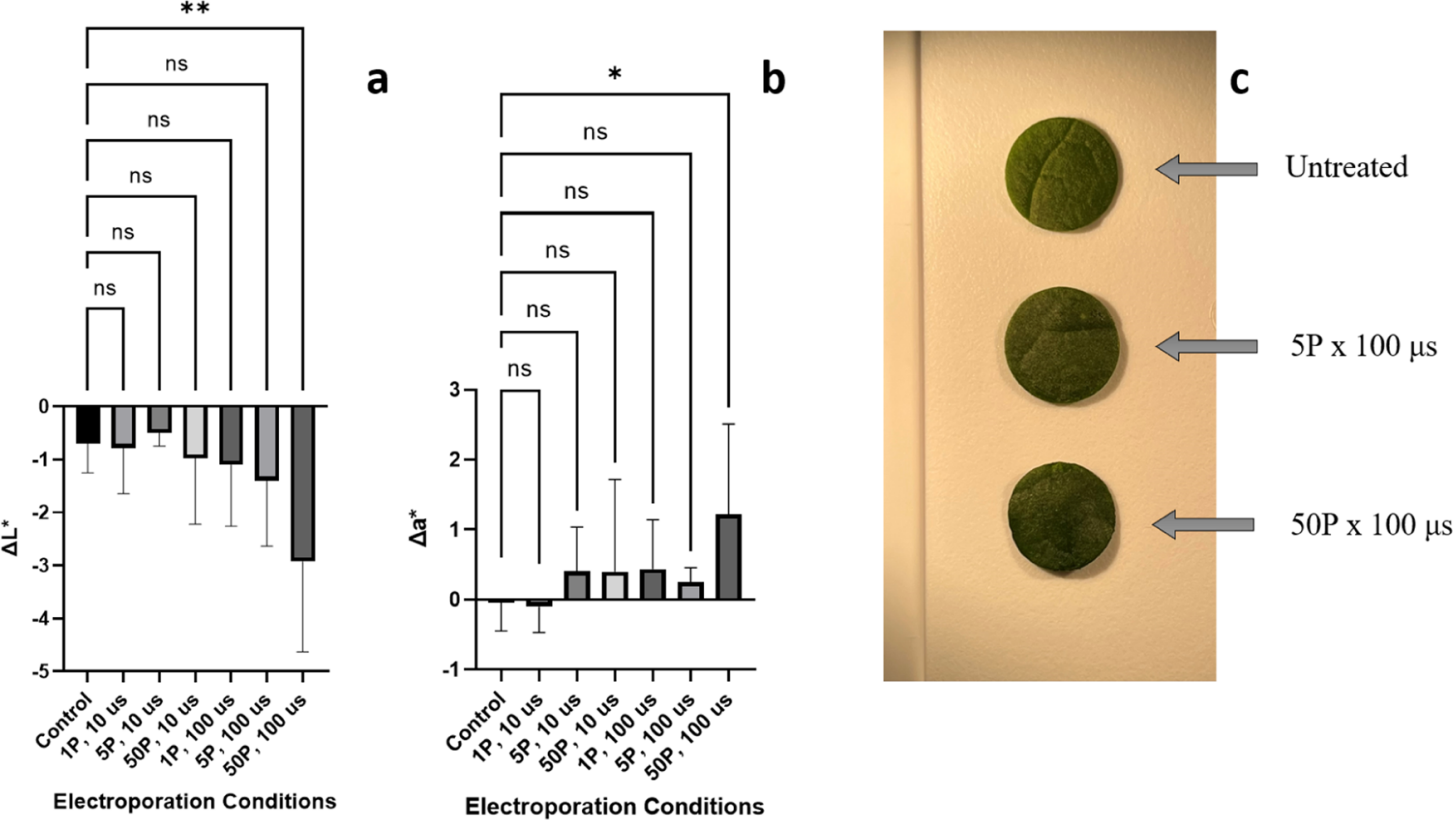
Differences in L* values after exposure for each parameter (a), differences in a* values after exposure for each parameter (b), and representative images of 3 conditions showing the darkness gradient with increasing intensity (c). Statistical significance from the one-way ANOVA results is represented as (ns) ≥0.05, *P ≤ 0.05, **P ≤ 0.01, ***P ≤ 0.001, ****P ≤ 0.0001. Error bars indicate 95% confidence intervals.

Changes in both L* and a* values are significantly different compared to the controls only for the 50P × 100 μs samples (Figure 5). For this treatment condition, the average change in L* is −2.9 ± 1.7 at a 95% confidence interval, compared to the controls’ average change of −0.7 ± 0.5 at a 95% confidence interval, indicating a darker color of the samples with that level of treatment. The 50P × 100 μs samples’ a* differences are 1.2 ± 1.3 at a 95% confidence interval, compared to the controls’ average change of 0.0 ± 0.4 at a 95% confidence interval. This increase in a* for this treatment condition indicates a less green coloration.

### 3.4 Viability

**Figure 6:**
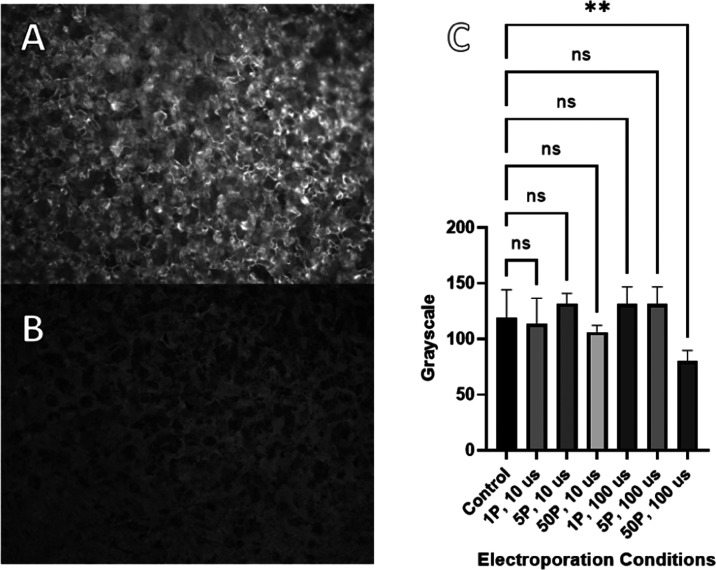
Microscopic image of (a) untreated, FDA-stained spinach cutouts and (b) spinach cutouts exposed at 50P × 100 μs. (c) Grayscale for each condition from fluorescence microscopy. Statistical significance from the one-way ANOVA results is represented as (ns) ≥0.05, *P ≤ 0.05, **P ≤ 0.01, ***P ≤ 0.001, ****P ≤ 0.0001. Error bars indicate 95% confidence intervals.

Fluorescence microscopy images reveal no significant differences in overall image mean grayscale values between treated samples compared to controls except for the 50P × 100 μs samples (Figure 6), wherein lower fluorescence intensity indicating cell death is observed.

### 3.5 pH Changes

**Figure 7:**
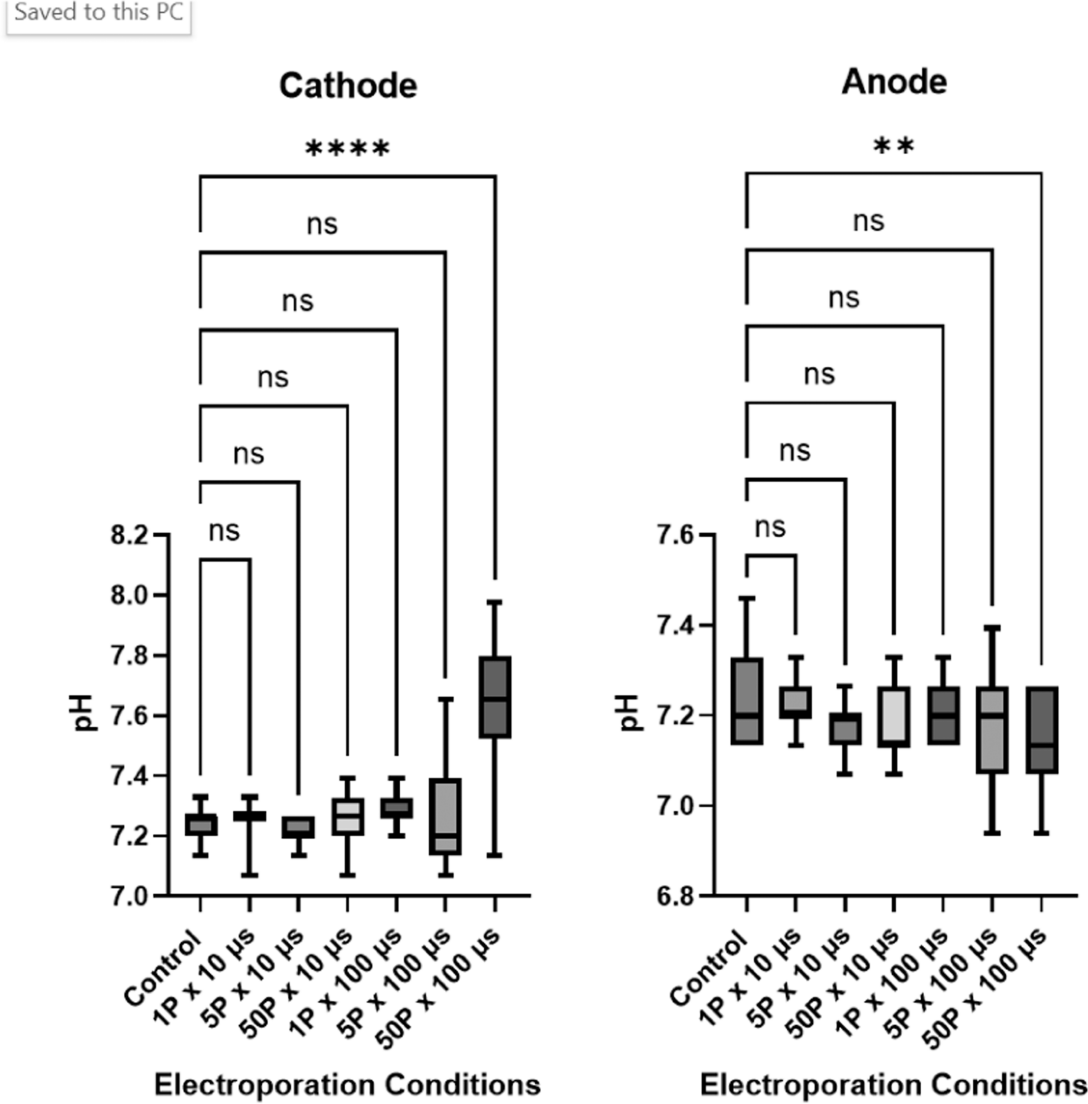
Box and whisker plots (min to max) at the anode and cathode after PEF exposure.

Final pH values are recorded in Figure 7 for the cathode and anode, respectively. Only the 50P × 100 μs samples display significant differences compared to the untreated samples at both the cathode and the anode. At this condition, alkalinization occurred at the anode (7.23 ± 0.03 to 7.63 ± 0.15) vs acidification at the anode (7.25 ± 0.06 to 7.14 ± 0.06).

### 3.6 Temperature Change

Temperature is predicted to rise from the initial ambient temperature of 20 to ∼24.5 °C at the end of an exposure of 50 pulses of 100 μs duration. Representative graphs from the simulation can be found in Figure S6.

## 4 Discussion

### 4.1 Spinach Quality

EIS in two-electrode mode can induce error when assessing the impedance of biological samples, especially at lower frequencies, due to electrode polarization (EP).^24−26^ EP occurs under the presence of an electric field, wherein an ionic double layer forms between the interface of the electrodes and a conductive fluid. This electrical double layer (EDL) has an associated impedance.^27,28^ The EDL impedance creates a false sense of increased impedance at lower frequencies.^27,29^

Because of this, in terms of magnitude, impedance ratios are most likely lower than what is reported. The effects of EP can be noted when comparing the 5P × 10 μs and 5P × 100 μs samples, in which the impedance ratio is lower for the higher intensity, longer pulse duration exposure at higher frequencies but not at lower frequencies, where EP would be the most prominent. For 50P × 10 μs and 50P × 100 μs, the impedance drop from electroporation dominates EP at lower frequencies. EP may not be prominent enough for the 1P × 10 μs and 1P × 100 μs samples to dominate, and so, a crossover in the middle of the frequency spectrum does not occur.

Lower impedance ratios at higher intensity exposures for a singular frequency have been documented for Thai basil^6^ and rucola leaves.^21^ Pore size and number will be larger with longer pulse durations^30^ and higher intensities,^18^ leading to greater ion leakage. After exposure, a greater decrease at the lower end of the frequency spectrum is expected because lower frequencies are unable to pass through an intact membrane.^25^ The cell membrane no longer presents resistance to electrical current in the higher MHz frequency range,^31^ and so, impedance ratios begin to reach similar values at the higher end of the frequency spectrum, regardless of exposure intensity. The greater decrease in resistance of the membrane, in addition to the greater leakage of ions from bigger and more abundant pores causing increasing buffer conductivity,^32^ leads to this decrease in impedance.

The double-shell model shows that changes for the electrical properties of the membrane are much more prominent than those for the inner components of the cell. Slight differences are seen for the vacuole and cytoplasm, but there is a large change, relative to other conditions, at 50P. With subsequent pulses, pores will be larger and more abundant. As the resistances of the plasma membrane and cell wall drop, more energy can be delivered to the intracellular contents of the cells, allowing for greater changes to occur.

The cell membrane can be treated as a capacitor^19^ that will charge when exposed to PEFs^33^ and is observed as such, increasing with higher pulse numbers and duration. The driving force for changes in electrical properties begins with the membrane and wall, wherein other contents can be affected once this barrier is penetrated. Similar to the *Z*_mod_ ratios, the magnitude of the resistances and capacitances may be higher than in actuality, with error due to the occurrence of EP, but the trends are still valid.

Electropores formed by electroporation vary in size^30,34^ and reseal at varying rates^30,35^ in a manner dependent on exposure intensity and the ratio of external solution conductivity to intracellular conductivity. While resealing times vary, plant and mammalian cells have been reported to fully repair in the order of seconds to minutes.^34,36,37^ The contents of a spinach leaf consist of almost 92% water by mass.^14^ With the presence of electropores, and increased permeability or electropermeabilization,^37^ there is potential that water could be outflowing from the leaves, similar to how PEFs are often used to extract juices from food products.^1^

While pore formation is evident from all exposures from the impedance changes, and although averages trend toward greater color changes with longer pulse durations, only the 50P × 100 μs condition induced significant changes in quality, in terms of weight and color. EIS has been shown to be correlated to cell death, where smaller changes in impedance are representative of a larger change in viability.^38^ The 50P × 100 μs parameter displays a clear difference in impedance postexposure in comparison to other samples. This condition was also the only one to display significant inactivation to the plant cells in accordance with the FDA fluorescence intensity. From the parameters evaluated in this study, it is evident that pore formation in itself is not the cause for decreased quality of leaves. Reversible pores may be too small or not open for long enough before resealing to see any large alterations. Rather, cell death, or full membrane rupture from irreversible electroporation, is required to change several physical characteristics of the leaves.

The small chloroplasts, containing chlorophyll that causes the green appearance in spinach leaves, are inefficiently damaged to release the pigment from PEFs even at much higher field strengths.^39^ It is therefore unlikely that the extraction of chlorophyll is the reason for a change in L* and a* values.

Natural color change (over time) of plant tissues has also been observed with a significant change in water content, as seen in litchi^40^ and rambutan.^41^ Since the total mass of spinach leaves is 92% water,^14^ and with the 50P × 100 μs condition about 16% of mass is lost after exposure, a considerable amount of water is extracted as a result of that level of PEF exposure. This could have induced browning, reducing the “greenness” and altering the luminescence. Color has been altered by PEF exposures in both apples and carrots,^42^ both of which do not have chlorophylls as their dominant pigment. This could either indicate that water loss (a commonality among the foods) causes discoloration or that different pigments are affected similarly by PEFs.

It is expected that further discoloration would occur over time both due to water loss and overall loss of cell integrity.^43^ The exact mechanisms underlying changes in appearance have not been determined within the confines of this study. The extracted mass also induces shrinkage (wilting) of the leaves, another major sign of bad spinach.

Discoloration can also occur because of the conversion of chlorophyll^43^ due to the degradation to pheophytin from magnesium ion removal.^44,45^ Chlorophyll is relatively sensitive and can be converted because of several factors, including changes in pH and temperature.^45^ On a scale from low to high, chlorophyll stability relative to temperature changes is considered to be “moderate”.^46^ Although a 4.5 °C increase in surrounding temperature is not sufficient for degradation in itself, its influence with other changing factors can play a role. Chlorophyll is generally considered to be stable between pH values of 3.5 to 5.0.^46^ On a scale from low to high, chlorophyll stability relative to pH changes is considered to be “low”.^46^ The higher intensity from the 50P × 100 μs condition could induce greater uptake in Gomori buffer, differing from the natural pH of raw spinach.

pH changes in the filter paper, and therefore in the solution surrounding the spinach, are a product of electrolysis, one of the most likely reactions to occur from PEF treatment.^47^ Decomposition of water into hydrogen gas at the cathode creates additional hydroxide ions, and decomposition into oxygen gas at the anode leaves a greater concentration of hydrogen ions, resulting in the respective alkalinization and acidification.

Immediate effect on viability is a result of the electric field and not because of electrolysis.^48^ However, the changes in pH and generation of new chemical species may create a harmful environment for surrounding cells.^49^ Electrolysis can solubilize the solid metals in the electrode.^50^ The deposition of metal ions onto the leaf surface can raise toxicity concerns and may have adverse effects on color and flavor.^47^ Because significant electrolysis occurs with the 50P × 100 μs samples, it is possible that secondary reactions induce the observed color changes. Anthocyanin, a pigment found in red cabbage, experiences electrochemical degradation from PEF treatment.^51^ PEF quality parameters have been examined for beer, however, the only noticeable change is of taste but not appearance.^52^

Regardless of food quality changes, limiting electrolysis is necessary for avoiding high concentrations of metals in the solution and extending the lifespan of the electrodes.^53^ This can be accomplished by using electrodes with greater resistivity to electrochemical reactions like titanium and platinized titanium or by using shorter pulses, such as 10 μs.^53^ In addition, medium with greater resistance can limit the current density, on which electrolysis is also dependent.^54^ Electrochemical reactions do not occur until the charging of the double layer reaches a threshold potential (∼1–2 V) and is proportional to double layer capacity but inversely proportional to current density.^53^

Pulse duration has a greater effect on metal ion release compared to applied voltage.^55^ Iron release from stainless steel electrodes varies for different applications. Rodaitė-Riševičienė et al. observe >0.5 mM increase in iron concentration from a 2 ms pulse at 1.2 kV/cm using S.S. electrodes.^56^ van Wyk et al. find metal ion concentrations “well below dangerous levels” after applying 1.7 μs pulses at 53 and 34 kV/cm to wine using S.S. electrodes.^57^ Altunas et al. monitor metal ion release at several pulse durations (0, 66, 105, 131, 157, and 210 μs) and field strengths (0, 17, 20, 23, 27, and 30 kV/cm) and see no significant levels of ion migration at any of these conditions.^58^ The differing results implicate the importance of pulse width and its role in electrolysis. Metal ion concentration has not been quantified in this study. But in the absence of pH change, and therefore the limit of electrolysis, for all samples with unchanged quality, it is reasonable to assume that a significant amount of ions have not leached into adjacent solutions. The correlation between electrolysis, metal ion solubilization, and spinach quality should be investigated in future works because of the significant alteration in pH for the 50P × 100 μs condition. Varying field strengths should also be investigated, as a greater number of pores can be formed with shorter pulse durations.

## 5 Conclusions

Pore formation in spinach cell plasma membranes is evident at low-intensity treatments, but intracellular components do not start to exhibit effects from PEF exposure until the cell membrane barrier has been bypassed by lower frequency components of the pulse exposures. Despite the greater formation of pores with greater specific energy delivered, discoloration and mass decrease are not observable until cell death occurs. It is possible that the reversible pores formed are not large, abundant, or open for long enough to release an amount of water from the spinach to inure drastic effects. Rather, when membrane rupture is observed, and the membrane loses its semipermeable barrier function, contents are able to exit the confinements of the cell and alter food product quality thereafter. The only condition with reduced quality postexposure experiences both loss of viability and electrolysis. Both must be avoided in order to maintain structural integrity and safe consumption after PEF treatment. Reversible pores can be formed with shorter pulses of larger quantity, evident by EIS, which also avoid electrolysis, such as the 50P × 10 μs used in this study. Careful selection of parameters to achieve enhanced permeability and maintain product quality is important for the application of enhanced subsequent processes and for maintaining consumer satisfaction.

## Supporting Information Available

pH analysis (including representative images); EIS spectra (including Cole–Cole and phase angle plots); and COMSOL multiphysics model and simulation graphs (PDF)

## Abbreviations

PEF: pulsed electric field
*R*_w_: resistance of the cell wall/membrane
*R*_cs_: resistance of the cytoplasm/symplasm
*R*_v_: resistance of the vacuole
*C*_cm_: capacitance of the cell membrane
*C*_vm_: capacitance of the vacuolar membrane
FDA: fluorescein diacetate
*Z*_mod_: total Impedance
ω: frequency
Δ: change in
τ_m_: charging time
TMV: transmembrane voltage
EIS: electrochemical impedance spectroscopy
EP: electrode polarization
EDL: electrical double layer
