# Supplementary material for: Threshold Microsecond Pulsed Electric Field Exposures for Change in Spinach Quality
Source: ACS Omega. 2023 May 24;8(22):19833–42. doi: 10.1021/acsomega.3c01454 (PMC10249097; doi:10.1021/acsomega.3c01454)
Supplement: Supplementary file 1 — ao3c01454_si_001.pdf [file ao3c01454_si_001.pdf]

SUPPORTING INFORMATION

Threshold Microsecond Pulsed Electric Field Exposures for Change in Spinach Quality

Zachary Rosenzweig[1,*], Abigail Martin[1], Colin Hackett[1], Jerrick Garcia[1], Gary L. Thompson[1,2,*]

[1]Rowan University, Department of Chemical Engineering, Glassboro, NJ 08028, USA
[2]Lead contact
*Correspondence: rosenz26@students.rowan.edu (Z.R.), thompsong@rowan.edu (G.T.)

This document contains the following supplemental information and tables:

- Standard curve for conversion of hue to pH
- Representative images from pH readings
- Graphical representation of double-shell model analysis of EIS readings
- Cole-Cole plots from EIS readings for before and after PEF exposure
- Phase angle plots from EIS readings for before and after PEF exposure
- COMSOL Multiphysics model simulation of Joule heating during 100 microsecond pulsed electric field (PEF) exposure

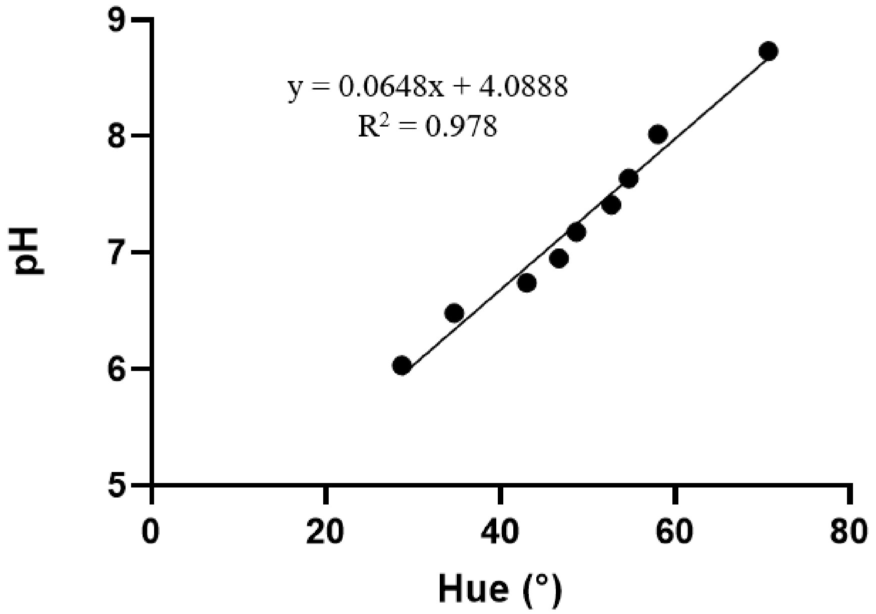

Figure S1. Standard curve for conversion of hue to pH

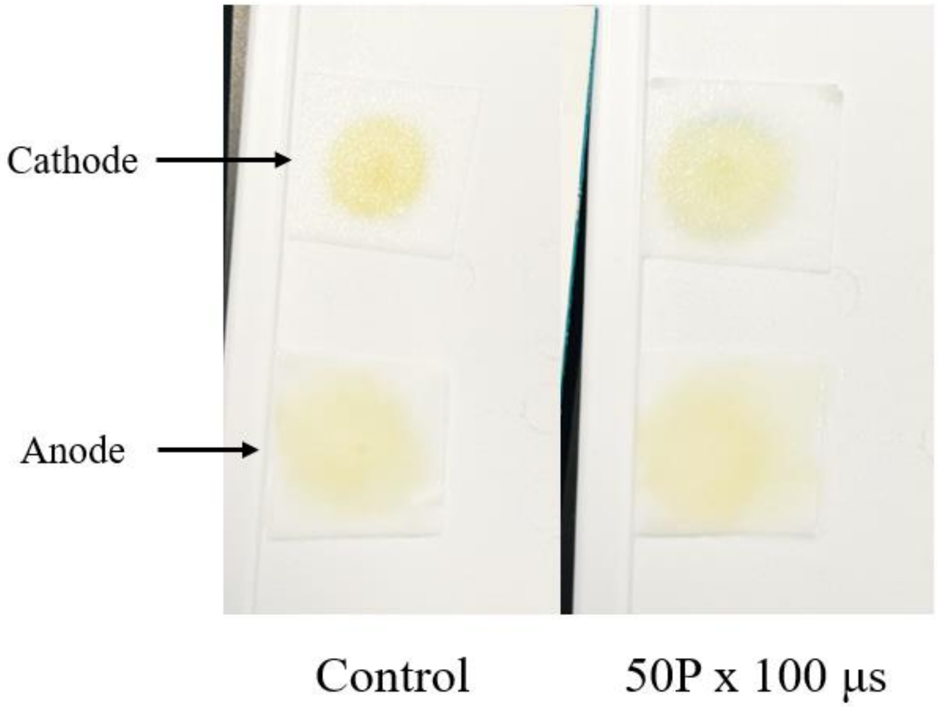

Figure S2. Representative images from pH readings

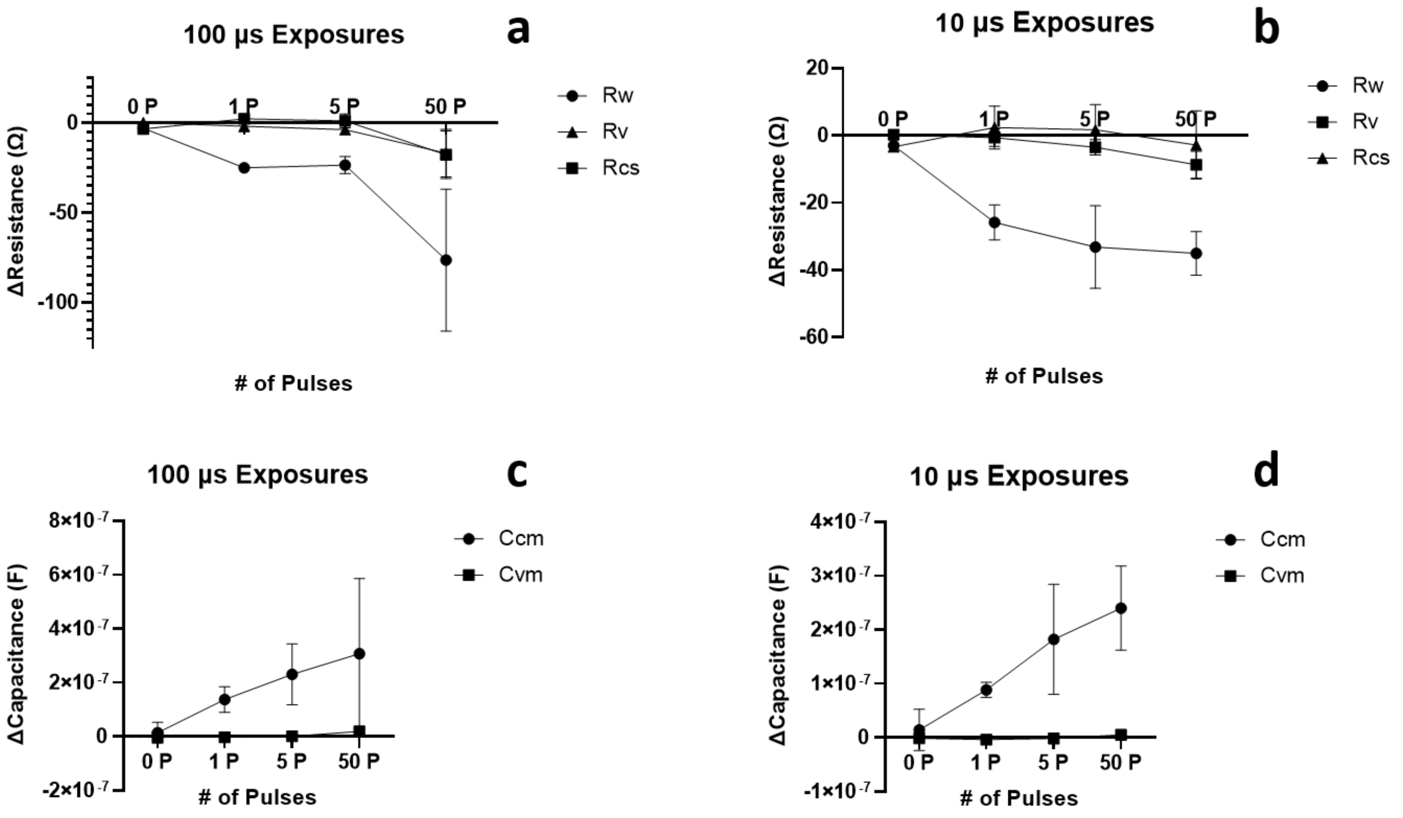

Figure S3. Plotted difference in resistance values from the double-shell model for 100 µs (a) and 10 µs (b) exposures, and difference in capacitance values from the double-shell model for 100 µs (c) and 10 µs (d) exposures. Error bars indicate 95% confidence intervals. Error bars smaller than the data points are not shown.

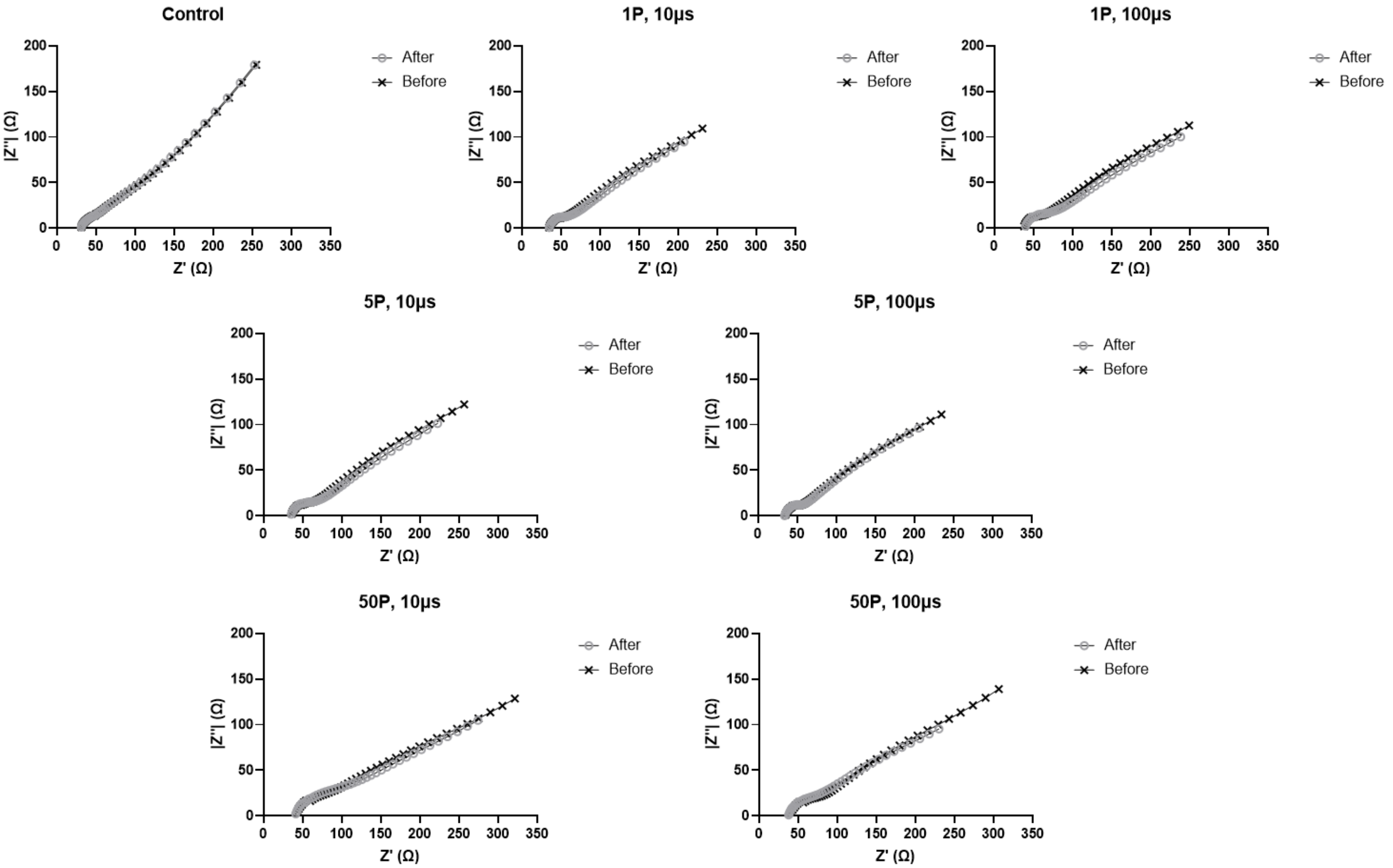

Figure S4. Cole-Cole plots for before and after PEF exposure for each parameter

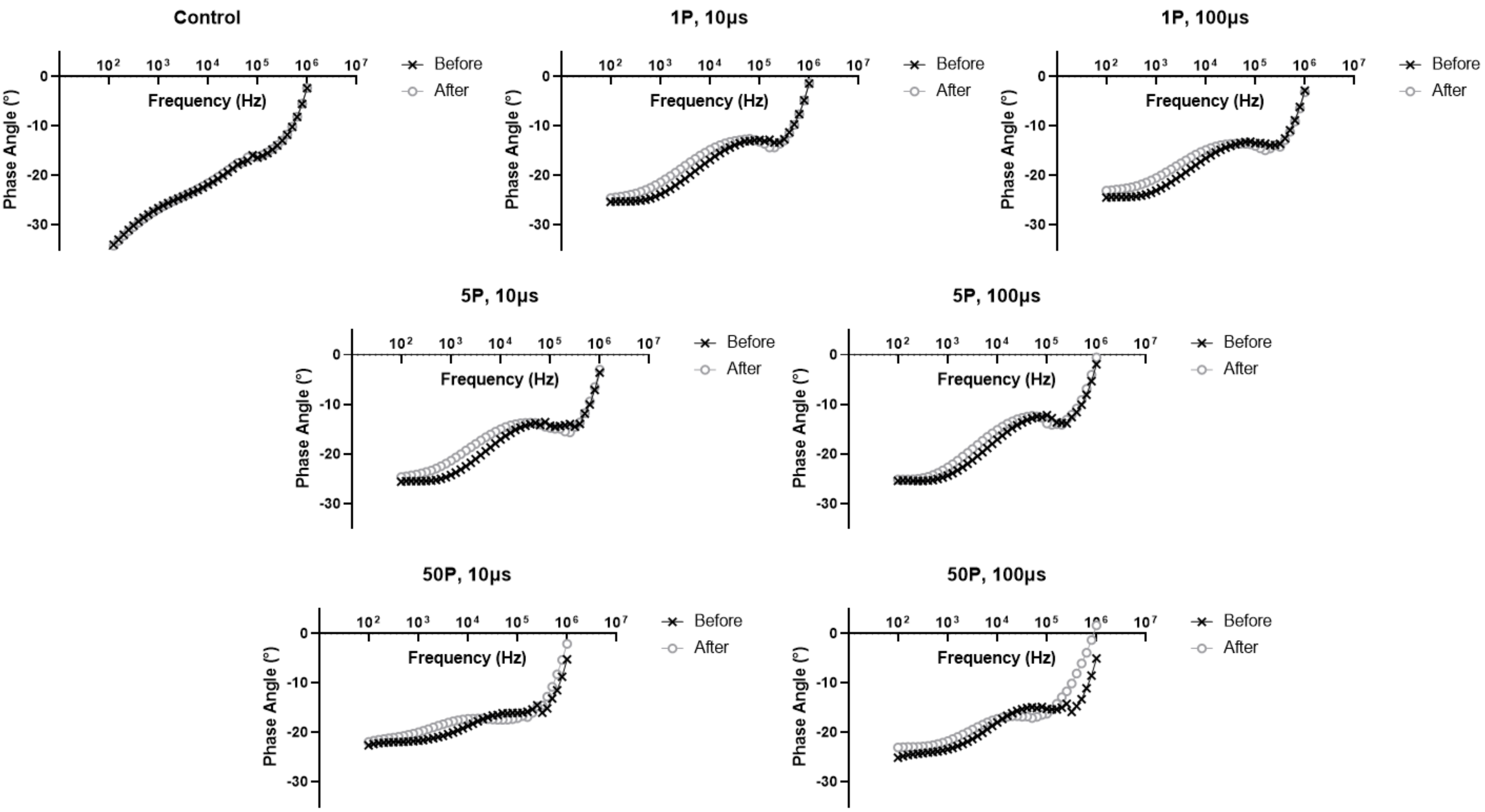

Figure S5. Phase angle before and after PEF exposure for each parameter

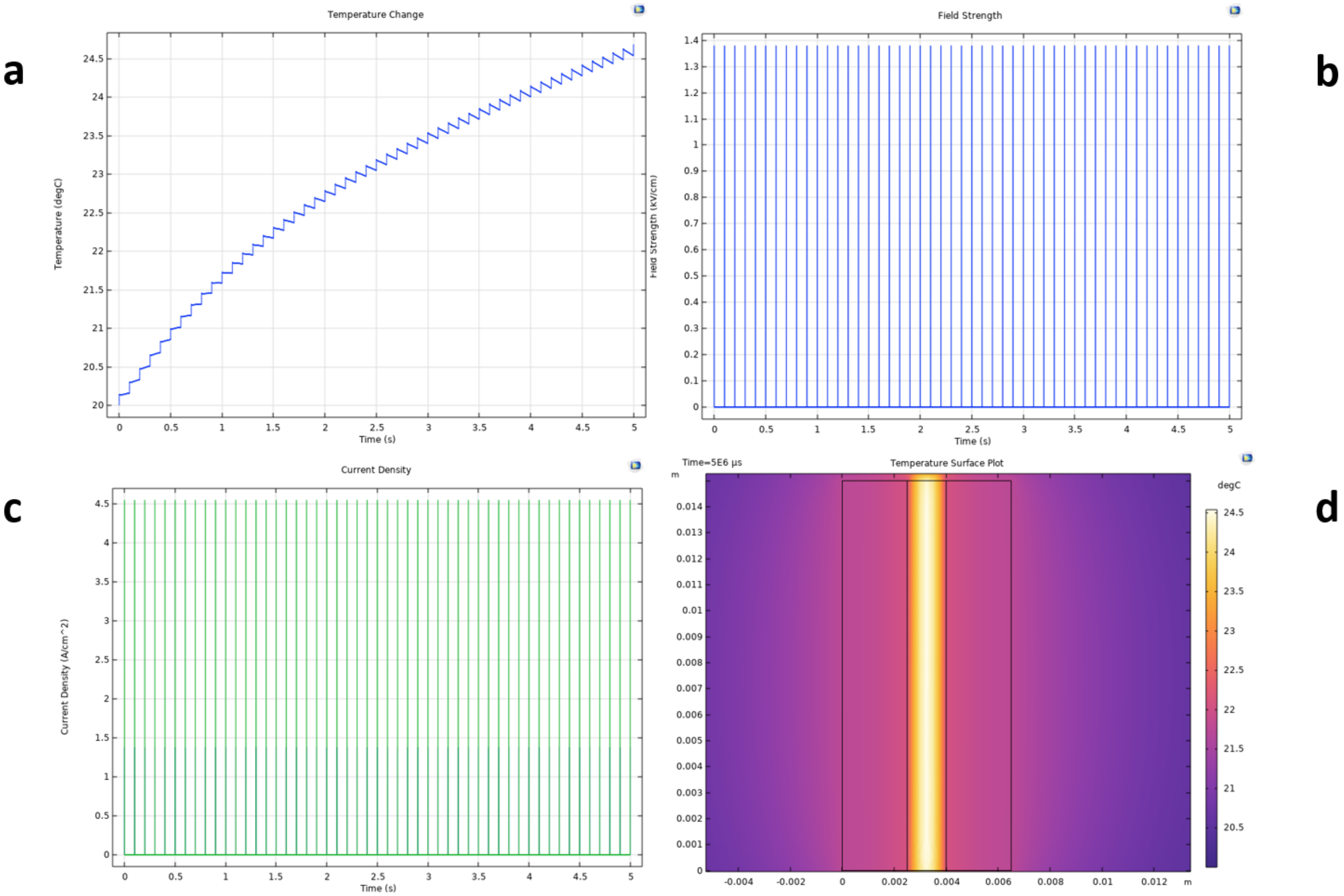

Figure S6. Temperature change vs. time in the center of the electric field (a) field strength vs. time between the electrodes (b) current density vs. time between the electrodes (c) surface plot of temperature at the end of PEF treatment (d).
